# Biomarkers of stress resilience: A review

**DOI:** 10.1016/j.nsa.2024.104052

**Published:** 2024-02-28

**Authors:** Pasquale Paribello, Igor Branchi, Aurelia Viglione, Giulia Federica Mancini, Maria Morena, Patrizia Campolongo, Mirko Manchia

**Affiliations:** a Unit of Psychiatry, Department of Medical Sciences and Public Health, University of Cagliari, Cagliari, Italy; b Unit of Clinical Psychiatry, University Hospital Agency of Cagliari, Cagliari, Italy; c Center for Behavioral Sciences and Mental Health, Istituto Superiore di Sanità, Rome, Italy; d Department of Physiology and Pharmacology, Sapienza University of Rome, Rome, Italy; e Neuropharmacology Unit, IRCCS Fondazione Santa Lucia, Rome, Italy; f Department of Pharmacology, Dalhousie University, Halifax, Nova Scotia, Canada

## Abstract

The complex dyadic interaction of stress and resilience has received growing attention as a promising avenue for informing new diagnostic and prognostic models for human health. In this review, we present a selection of some of the most relevant data on translational models and biomarkers of stress and resilience in the field of mental health. Several critical aspects concerning the preclinical and clinical model development are addressed. The distance between preclinical and clinical disease models has widened with time across all fields of medicine, with psychiatry presenting additional hurdles represented by the inherent heterogeneity of the studied phenotypes. Capitalizing on technological advances in developing and consolidating sound theories for stress-resilience interaction models represents a promising avenue, possibly endowed with greater ecological validity compared to the sole socio-psychological assessment. Instrumental in advancing the field will be an increased level of integration between preclinical and clinical researchers' efforts in developing translational biomarkers, aiming to elucidate better the interindividual heterogeneity in the impact of stress exposure on individuals’ health and behavior.

## 1 Introduction

**Fig. 1:**
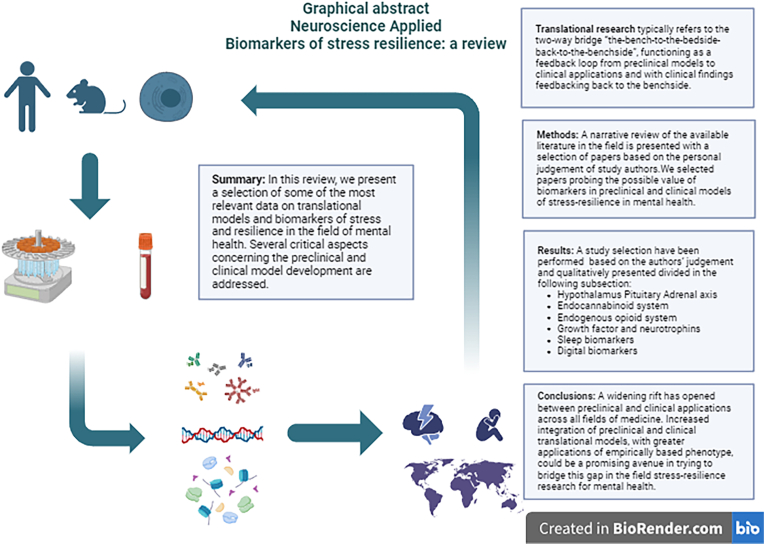
Fig. 1 - Graphical description for the two-way-bridge between preclinical, clinical models.

Translational research typically refers to the development of experimental models that capitalize from advancement in basic sciences acquisitions in the development of new treatments or clinically viable biomarkers: in other words, the two-way bridge “the-bench-to-the-bedside-back-to-the-benchside”, functioning as a feedback loop from preclinical models to clinical applications and with clinical findings feeding back to the benchside (Cohrs et al., 2015). Despite growing interest and significant investments across various branches of medicine, a progressively widening rift has opened between preclinical and clinical models, resulting in relatively modest clinical impact of basic scientific advancements (Seyhan, 2019). In the mental health field, additional hurdles must be overcome, not least by refining the phenotypic definitions of the trait(s) under study. With the term stress, we typically refer to any physical or psychological stimuli that may disrupt homeostasis. The physiological and behavioral response related to the said stressors is called stress response (Chu et al., 2023). However, finding a satisfactory definition for such a complex range of phenomena may represent a difficult proposition (Del Giudice et al., 2018). In the mental health field, the current literature focuses on the cognitive part of stress, defining it as a threat perceived by the organism as impossible to control or predict (Del Giudice et al., 2018). Arguably, a more expendable definition in terms of translational research may define stress more broadly as a failure of the organism to control a fitness-critical element, which may originate either externally or internally (Del Giudice et al., 2018), and defining fitness as the ability of an organism to survive and reproduce (Orr, 2009). Trying to elucidate the underpinnings of the association between stress and relevant clinical outcomes appears similarly complicated as trying to grasp an appropriate definition for stress itself. On the one hand, the association between stress exposure and the development of mental health disorders has been well documented over the years, with some lines of evidence suggesting a dose-response association between stressors (such as traumatic experiences, i.e. violence, natural catastrophes, etc) and the risk of developing mental disorders (Murphy et al., 2022). On the other hand, the vast majority of the studies present a variety of elements that may complicate their interpretation, such as the possibility of recall bias in trauma reporting (Newbury et al., 2018), the pre-existence of risk factors preceding traumatic events (DiGangi et al., 2013; Spinhoven et al., 2015), the observation that the vast majority of individuals exposed to traumatic events do not go on to develop a mental disorder (Lewis et al., 2019). These elements suggest that factors other than the traumatic events per se may influence the individual's capacity to cope with stress. Indeed, part of the interindividual variability in being liable to psychopathology after stress exposure appears to derive from subjective predisposing elements. In an apparent contradiction with the “sensitizing hypothesis” that would regard trauma exposure solely as a possible cause of psychopathology, a sizeable body of literature has been devoted to the possible strengthening of psychological resources following trauma exposure, the so-called “steeling” effect (Kok et al., 2021). In this context, resilience is aptly defined as the psychological immune system, the subject's innate capacity to overcome stressors (Paris and Paris, 2022). On a global scale, being exposed to traumatic events may represent the norm rather than the exception. According to this paradigm, accumulating negative experiences may lead in certain settings to a progressively smaller detrimental effect from further trauma exposure (Kok et al., 2021; Sharpley et al., 2021). On the same note, “post-traumatic growth” refers to a positive change in a subject's attitude following the struggle with a life-changing event (Sawyer et al., 2010). Receiving a diagnosis of a life-threatening condition such as cancer or HIV may represent a trauma satisfying the A criterion for PTSD according to the Diagnostic and Statistical Manual of Mental disorder IV or 5th edition under specific circumstances (e.g., disease representing chronic stress source, critical illness either due to the direct consequences of the disease or complications related to the treatment). PTSD prevalences in these populations ranges from 5 to 35% and 30–64% in these populations, respectively (Sawyer et al., 2010). Similar to what is observable in association with other types of trauma exposure, past reports described positive changes in attitude following the traumatic event in 59–83% of people living with HIV and in 60–90% of cancer survivors (Sawyer et al., 2010). From a biopsychosocial perspective, disorders may be defined as the inextricable result of the mutual interactions of biological, psychological and environmental factors (Wade and Halligan, 2017), with each element's contribution being inalienable from each other and understandable only in the context of their dynamic association. Personality traits represent a relatively stable set of heritable features comprising ways of thinking, behaving and feeling (Briley and Tucker-Drob, 2014). Despite early evidence suggesting otherwise, more recent reports suggest that there may be relatively consistent personality patterns even among different countries, with deeper personality trait differences within countries rather than between countries (Kajonius and Mac Giolla, 2017). Therefore, personality trait assessments may represent a useful platform for advancing our understanding of part of the interindividual heterogeneity in stress resilience. Among them, psychological traits such as neuroticism have been extensively studied in this context (Ormel et al., 2013). Depending on the considered environment, from an evolutionary perspective, presenting a high neuroticism level may be associated with a prominent stress response and, therefore, represent an adaptive trait in situations where it might be preferable to be more vigilant and risk-averse (e.g., taking extra measures to avoid predators rather than assuming their absence -Paris and Paris, 2022; Sharpley et al., 2021). However, neuroticism traits might still be associated with a detrimental effect in other settings, such as a higher risk of developing trauma- and anxiety disorders. The net effect of environmental exposure may also change with time and depending on the considered sociocultural milieu. In line with this concept is the observation that despite the apparent concordance between exposure level and lifetime risk of PTSD between individuals and groups, the same does not apply to the comparison between countries (Dückers et al., 2016). On a global level, PTSD prevalence is similar for countries with low exposure to trauma and low vulnerability compared to countries with high exposure and high vulnerability (with vulnerability defined as a composite index condensing socioeconomical and environmental circumstances), whilst countries with low vulnerability and high exposure have on average three times as high PTSD prevalences (Dückers et al., 2016). A similarly paradoxical association related to the vulnerability-exposure interaction has also been described for the prevalence of major depressive disorder across countries (Dückers et al., 2016). This irreconcilable contradiction is more clearly understandable considering different levels of access to care, different sociocultural environments, different diagnostic thresholds, and different levels of expected exposure to traumatic events. In other words, in contexts where trauma is more common, the social meaning of trauma may change drastically, and therefore, its result in terms of clinical outcomes (Paris and Paris, 2022). From a biological point of view, when discussing the development of translational models in psychiatry, several elements need to be considered to assess the difficulties encountered by researchers in the field. Establishing a direct association between a set of subjective experiences as reported by an examined subject and interpreted by an examiner and using the said symptoms as a biologically-based diagnosis is a significant paralogical leap. This phenomenon may partly explain the difficulties in developing clinically viable, diagnosis-based biomarkers in the field of psychiatry. As a comparison, dyspnea, a subjective negative experience, may depend on various causes and is now recognized as being associated with various pathophysiological mechanisms and recognizes very different treatment protocols (Fukushi et al., 2021). At the beginning of the 20th century, great attention was devoted to fever classification, only to later recognize the lack of clinical or biological utility of such paradigms, as fever represents merely a non-specific reaction to a wide range of different conditions rather than each form (e.g., blackwater fever, yellow fever) representing distinct pathological conditions per se (Lilienfeld and Treadway, 2016). Numerous different laboratory and instrumental tests may assist clinicians in defining some of its causes, and still, to this day, trying to formulate an appropriate diagnosis at the singular patient level may represent a very significant challenge for clinicians, especially considering the complexity and variety of the possible underlying causes. Currently, fever of unknown origin, variedly defined as fever lasting more than two-three weeks that remain undiagnosed after an extensive diagnostic panel, still represents over half of the total cases (Haidar and Singh, 2022). That being said, if possible, in psychiatry, the diagnostic process is even more complicated than in other areas of medicine, as there is no external validator outside of the clinical assessment for primary psychiatric diagnoses (i.e., not secondary to general medical causes, exposure to exogenous substances, etc - Lilienfeld and Treadway, 2016; Haidar and Singh, 2022 ). Considering the significant heterogeneity in reported clinical outcomes (Bryant et al., 2023; Maj, 2015), inherent heterogeneity in the psychiatric diagnostic process and uncertain biological correlations (Schmaal, 2023), the low reliability of diagnoses among different providers (Vanheule et al., 2014), and the presence of shared genetic and neurobiological traits among different diagnoses (Schmaal, 2023), the possibility of developing biomarkers that may ultimately aid clinicians either in the diagnostic process or in prognostic stratification may represent a daunting task. In this sense, the possibility of capitalizing on technological advances for the development and consolidation of sound theories for stress-resilience interactions based on empirically based phenotype profiling represents a particularly promising avenue, possibly endowed with greater ecological validity as compared with the sole socio-psychological assessment. Instrumental in this endeavor is the increasing integration of translational biomarkers, aiming to elucidate better the interindividual heterogeneity in the impact of stressors on the individual's health and behavior (Harrist and Gardner, 2019). This represents a particularly active area of research requiring the contribution of various professionals and the integration of data deriving from numerous areas of research. For example, animal model behavior readouts do not represent good proxies for complex human behaviors, especially when the assessed outcomes in such preclinical models represent the product of highly controlled and short-lived tests. Even accounting for the inherent difficulties and, maybe, the impossibility of capturing the complexity of the human subjective experiences explored in mental health sciences within animal models, even the most refined animal models hardly encompass what might represent the influence of socioeconomic impoverishment on human mental health (Shemesh and Chen, 2023). Arguably, nuanced models comprising semi-natural monitoring might represent a step forward in producing more ethologically valid models of translational value, possibly representing evolutionary-conserved paradigms more representative of the human biology (Shemesh and Chen, 2023). On the other hand, similarly, developing viable preclinical models for the relatively ill-defined borders of human pathology in mental health might represent an equally difficult proposition. Hopefully, having a wide, shared perspective across different disciplines for this complex transition will be mutually beneficial in advancing the field and further enrich our understanding of complex endophenotypes relevant for translational research. In this context, we propose a review of studies describing biomarkers research of preclinical and clinical models of stress and resilience in the field of mental health, with the purpose of offering a general overview for some of the most relevant papers published in this complex and everchanging landscape (see Fig. 1).

## 2 Methods

A narrative review of the available literature in the field is presented, with a selection of papers based on the personal judgement of study authors for the most relevant articles. To further enhance our review, we performed a literature review with the following search strategy on PUBMED/MEDLINE ‴biomarker' AND 'resilience' AND ('mental health' OR 'mental disorder' OR 'mental illness')" and focused on the following mental disorders: Mood disorders, Stress Disorders, Psychotic Disorders, Substance use/abuse/dependence. In the present project, we focused on papers reporting on original research probing the possible value of biomarkers in preclinical and clinical models associated with the concept of stress-resilience in mental health in terms of 1) predicting the development of a particular mental illness as defined according to current diagnostic categories, 2) treatment response, 3) anxiety or depressive symptoms, or other outcomes as defined by the relative study authors and deemed relevant. We analyzed only papers published from 2000 to 2023 (the last search was performed on the November 15, 2023). A non-systematic review was then carried out, with two authors (PP and MM) selecting the most pertinent papers according to personal judgment and following the stated criteria. PC, MM, GFM, IB focused on reports describing preclinical models. An extensive pearl-growing strategy was employed on pertinent reviews, books and other sources to further enlarge this research project's scope. A brief overview of the study content is then presented, subdivided into the corresponding paragraphs and depending on the considered biomarker category. A detailed discussion condensing the most salient results of the proposed selection is then proposed. Considering the significance of cultural and environmental factors at large in influencing the impact of trauma exposure and its association with psychopathology, we decided to include the country of origin for each of the studies described in the qualitative analysis.

## 3 Potentials and pitfalls of translational biomarkers in advancing research on stress and resilience

As previously defined, resilience is the ability to adapt positively in the face of adversity (McEwen et al., 2015). Resilient individuals are subjects experiencing a stressful/traumatic event that, after the first acute physiological response, do not develop psychopathologies (Feder et al., 2009). While conventional interventions often target symptom management, enhancing an individual's resilience holds promise as a therapeutic strategy for stress-related disorders (Kalisch et al., 2017). However, to do that, mechanisms as well as biomarkers of stress resilience need to be uncovered.

Animal models represent a key component in this context as they can reproduce similar alterations at the level of brain circuits, neurotransmissions, genes, and behaviors observed in patients with stress-related psychopathologies (D'Haenen et al., 2002; Yehuda and LeDoux, 2007). For instance, in rodent models, exposure to stress induces an exaggerated amygdala activation (Rosenkranz et al., 2010; Prager et al., 2016) and a hypoactivation of the hippocampus and prefrontal cortex (Whittle et al., 2010), which has also been found in patients with stress- and trauma-related psychopathologies such as anxiety and PTSD (Ormel et al., 2013; Gurven et al., 2014; Harrist and Gardner, 2019; Dückers et al., 2016; Fukushi et al., 2021; Lilienfeld and Treadway, 2016). Besides the documented similarities in the alterations of stress-sensitive brain areas, compelling evidence has been reported that resilience/susceptibility to stress-related disorders in humans and rodents share common molecular mechanisms (e.g., neural pathways and genes). It is well established that dysregulation of the HPA axis may lead to maladaptive mechanisms, resulting in the development of stress-related disorders (Juster et al., 2010). Evidence has shown that subjects suffering from stress- and trauma-related disorders and rodent models present overlapping alterations of the HPA axis and glucocorticoid receptor (GR) signaling (Daskalakis et al., 2013, 2014; Wen et al., 2015). Furthermore, altered *FKBP5* gene expression, which encodes a transcription factor that modulates GR function (Wochnik et al., 2005), has been identified in several animal models of psychiatric disorders, including depressive-like and PTSD-like models (van Doeselaar et al., 2023; Hä et al., 2021; van der Doelen et al., 2014; Criado-Marrero et al., 2017; Xu et al., 2017; Torrisi et al., 2021). Mirroring these alterations, variants of the *FKBP5* gene have been identified in human studies as biomarkers for stress-related disorders, such as anxiety, depression, and PTSD (Maul et al., 2020; van Zuiden et al., 2012; Michopoulos et al., 2015; Binder et al., 2008; Castro-Vale et al., 2016). As an example, dysregulation of the monoaminergic system is also involved in the vulnerability/resilience to develop stress-related disorders (Ryan and Ryznar, 2022) and overlapping alterations in humans and rodents have been documented in this regard. A polymorphism of the gene *SLC6A4*, encoding the serotonin (5HT) transporter (SERT), has been linked to the etiology of anxiety-related traits in humans (Mazzanti et al., 1998; Forstner et al., 2017) and *SLC6A4* knockout mice exhibit an anxious-like phenotype (Uher and McGuffin, 2008). These are only a few examples of common determinants of resilience/vulnerability to stress-related disorders identified in laboratory animals and humans, highlighting the translational value of using animal models to identify novel biomarkers.

Different experimental paradigms have been used in laboratory animals to model stress- and trauma-related disorders and to identify molecular and/or behavioral predictors of stress resilience (or vulnerability, Vanheule et al., 2014,–Linnman et al., 2011, Juster et al., 2010, Daskalakis et al., 2013, Daskalakis et al., 2014, Wen et al., 2015, Wochnik et al., 2005). Experimental strategies in laboratory animals focus on the initial identification of a given variable (e.g. early life stress, genetic differences) expected to induce a specific change in stress responsivity leading to the development of stress-related disorders following exposure to a stressor and then evaluate measurable behavioral or molecular markers to be related to the observed alterations. Alternatively, a different approach consists of the retrospective identification of biomarkers of stress resilience. In this latter approach, animals are stratified in resilient and susceptible populations based on their stress responsivity, allowing for unbiased identification of biomarkers based on the animals' global response (as previously described in Daskalakis et al., 2013). The availability of diverse animal models provides a solid platform for the rigorous identification and evaluation of numerous biomarkers to predict stress resilience and susceptibility to be translated and tested in the clinical environment. Vice versa, biomarkers of human patients can be easily tested in animal models for the identification of molecular mechanisms underlying the development and progression of stress-related disorders. Overall, the translational value of experimental paradigms lies in their ability to bridge the gap between preclinical and clinical research, contributing to the identification of biomarkers of stress vulnerability/resilience, which in turn allow for diagnostic precision and therapeutic innovation to treat and prevent the development of stress-related disorders in humans.

## 4 Results

The described search strategy yielded a selection of 443 single papers, screened by title and abstract from an unblinded researcher. After this first step, 172 full papers were then downloaded and assessed for relevance, with 32 papers representing either editorials or reviews. Most unique records assessed were considered either irrelevant or failed to report on the biomarkers of stress resilience and were excluded (n = 124). Therefore, the following sections for this review have been mainly developed by identifying additional sources either from selected bibliographies of the selected papers or from alternative sources based on the authors’ judgement.

### 4.1 Hypothalamus pituitary adrenal axis

Considering the potential biological association between stress response and HPA axis activation, great attention has been devoted to analyzing its association with neuropsychiatry disorders (Murphy et al., 2022). However, albeit frequently reported, HPA axis derangements are inconsistently observed, even within the same psychiatric condition (Murphy et al., 2022). Twelve of the selected papers reported on the possible worth of HPA axis-based biomarkers in mental health. The results of the qualitative analysis are summarized in Table 1. Six of them assessed the cortisol response through a variety of stress-response paradigms (Dajani et al., 2018; Agorastos et al., 2023; Feldman et al., 2013; Kerr et al., 2020; Lupien et al., 2022; Yirmiya et al., 2018), with only one comprising a follow-up assessment up to eleven months (Dajani et al., 2018). The remaining represented either a secondary analysis of previously published research (Smeeth et al., 2023), cross-sectional (Li et al., 2019; Barry et al., 2021; Petros et al., 2013; Li et al., 2021; Yirmiya et al., 2022) or retrospective study (Jang et al., 2022).

**Table 1:** Selection of paper reporting on clinical models of biomarkers involving the HPA axis.

Author, year	Study design	Sample size	Sample characteristics	Diagnostic category or clinical correlate	Main outcomes reported	Main results	Exclusion criteria	Country
Agorastos et al., 2023	Experimental study	14	- Adults (36.9 ± 2.3 y.o.) - Males = 35.7%	- non-psychotic, medication-free MDD (DSM-IV TR) - IDS minimum cut-off = 23/24 - HDRS minimum cut-off = 17/18	- Comparison between first-episode vs recurrent-MDD patients in repeated saliva (DHEA, CORT, SDHEA) and plasma (CORT, ACTH, CoP) HPA-related biomarkers in three days overnight challenge (i.e., following MET stimulation and DEX suppression)	- Recurrent-MDD ↓ salivary DHEA	- History of any physical or psychiatric co-morbidity (excluding hypothyroidism in the euthyroid state or arterial hypertension in normotensive state through medication) - Frequent use of any illicit substances or prescription, or over the counter medication - Actual menstruation, pregnancy, nursing or no adequate contraception - Psychotropic exposure in the last 8 weeks - Psychotic MDD history - BMI beyond 18–30 kg/m^2^ - Drinking >100 g alcohol/week - Current adverse life events - Transcontinental flights across >4 time zones in the last 4 weeks - abnormal basic laboratory test	GER
Alhalal and Falatah, 2020	Cross-sectional study	156	− 18-50 y.o. Women	- CAS - PCL-C - CESD	Comparison of HCC for IPV-exposed vs non-exposed women	IPV severity and resilience were associated with lower HCC	N/A	SAU
Baran et al., 2021	Cross-sectional study	7712	- Adults (51.7 ± 15.8 y.o., 56% female)	- SF6D	Probe the association of self-rated health with twelve physiological biomarkers considered as allostatic load index, including SDHEA	No association between mental health decline and the allostatic load index, as defined by the study authors.	N/A	UK
Dajani et al., 2018	Experimental study – prospective study	733	- Adolescents (12–18 y.o.) - Syrian refugee n = 411 - Jordanian non-refugee n = 322	- HI - PSS - CRIES-8	- Effects of structured, psychosocial intervention on HCC -Comparison of repeated HCC in three time points (basal, at the end of 2.8 weeks post-interventions, 11.3 months follow-up) for PTSD vs non-PTSD participants	- The intervention overall ↓ HCC by 1/3 - ↑ HCC in participants with hyposecretion - ↓ in participants with hypersecretion - No difference based on PTSD, gender, or resilience	N/A	JOR
Feldman et al., 2013	Experimental study	232	- Children 1.5–5 y.o., mothers 22.3–47.4 y.o. - n = 56 PTSD -n = 98 RE -n = 84 controls	- PTSD -DC 0 3R -Evocation of trauma associated with a fear paradigm (Lab-TAB adaptation)	Repeated child and mother SC and SAA before and after challenge and at recovery; comparison between PTSD and resilient-exposed groups	- PTSD children ↓ SC and SAA – higher levels of child withdrawal - RE children ↑ of SC and SAA – higher comfort-seeking - Controls children with low SAA, increased CT following challenge and decreased at recovery	- History of maltreatment or family violence; -Mothers reported physical or sexual abuse; -Child suffered severe motor vehicle or other major trauma other than war	ISR
Jang et al., 2022	Retrospective study	73	Adults (21–58 y.o.)	PROVE battery	SC and psychosocial factors between groups	- AUCg was positively correlated with depressive symptoms, anxiety and avoidance attachment, adverse childhood events, and mentalization problems. - PROVE groups ("good", "normal", and "cautious" risk groups for depression) presented different AUCg levels	- Physical diseases or substance exposure associated with MDD - Psychotropic medications for the last six months - Adrenal dysfunction - Major psychiatric disorder diagnoses - Severe physical diseases - Unable to read the consent form - Recent oral or cavity treatment	SKR
Kerr et al., 2020	Experimental study	203	- Hospital workers − 70% women	- BDI-II - ERIWQ - MBI	- Comparison of repeated diurnal SC measures between psychotropic-users vs non-users - SC reactivity after challenge (i.e., TSST)	Decreased SC reactivity to the TSST among psychotropic users	N/A	CAN
Li et al., 2019	Cross-sectional study	22	Pregnant women – PTSD-D n = 4; PTSD only n = 6; Resilient controls n = 12.	PTSD-D, PTSD	Repeated plasma levels of oxytocin and cortisol comparison among the three study arms (PTSD-D, PTSD, RC) during a 24-h protocol (blood drawn every 4 h for 24 h - n = 15) or 90-min protocol (blood drawn every 10 min for 90 min - n = 7)	Reciprocal levels of oxytocin and cortisol in all study groups	Smoking, more than 16 weeks gestational age, history of psychotic disorders, acute illness, molar pregnancy, multiple gestations.	USA
Lupien et al., 2022,	Experimental study	123	Adults (19–55 y.o.)	- Perceived Stress Scale - BDI-II -STAI-Y	Comparison for repeated SC and SAA secretion before and after challenge (TSST) between self-identified HS or ZEN participants	Chronic diseases (e.g., endocrine, cardiovascular, psychiatric)	No difference in SC or SAA despite variable psychological and socioemotional factors	CAN
Petros et al., 2013	Cross-sectional study	32 (subsample of wider, survey conventional sample N = 196)	- Adults (30 ± 10.9 y.o.)	- CD-RISC - GSE - LOT-R - STAI-Form-Y - CES-D-10 _WHO-5-WBI - ELSI	Correlation of single assessment for saliva CORT and SDHEA with clinical assessment	Positive correlation for SDHEA and resilience	- Previous or current psychiatric or physical illness - BMI >32 - Corticosteroid use - Regular over-strenuous exercise - Smoker/nicotine replacement - Major life event one month prior to enrollment	UK
Smeeth et al., 2023	Secondary analysis BIOPATH study	1600	Children (8–16 y.o.) with consenting caretakers	- CES-DC - CPSS - SDQ - WEQ	Evaluating the association of HCC and PRS with clinically assessed outcomes (i.e., risk of developing PTSD, depression or externalizing behavior problems)	N/A	- No association for PRS with depression, self-harm or neuroticism - HCC significant interaction for PRS and HCC with depression	LEB
Yirmiya et al., 2018	Experimental study	111	Adolescents – chronic trauma as mother-child interaction pattern – n = 58 exposed; n = 53 controls.	Trauma measure: CIB -Anxiety measure: SCARED	SC and IgA secretion between exposed vs non-exposed at baseline, 10 min after an "Etch a Sketch" paradigm and after 1,5 h	Trauma exposed had higher SC, higher IgA, had higher anxiety levels and collaborated less with mothers. Exposed mothers had higher SC and IgA and were less supportive during the task	N/A	ISR
Yirmiya et al., 2022	Cross-sectional	426	- LC n = 177 (Mage 9.3 ± 1.4 y.o.) - EA n = 111 (Mage 15.6 ± 1.2 y.o.) - LA n = 138, (Mage = 15.6 ± 1.3 y.o.)	- LC: DAWBA (Children assessment), PDS (maternal PTSD); - EA: CIB (behavioral observation), PCL-5 (maternal PTSD); - LA: DAWBA (children)	Mother and child HCC association with tested clinical assessments	- Trauma-exposed children had more internalizing disorder symptoms - Maternal exposure or sensitivity or symptoms appear to mediate continuity in internalizing psychopathology for the LC. Child HCC appeared associated with maternal PTSD	N/A	ISR

Abbreviations: ACTH - Adrenocorticotropic hormone; AUCg – Area Under the Curve with respect to ground: BDI-II – Beck Depression Inventory II; BMI – Body mass index; BSSS – Berlin Social Support Scale; CAN – Canada; CAS – Composite Abuse Scale; CD-RISC – Connor-Davidson Resilience Scale; CESD – Center for Epidemiologic Studies-Depression; CIB – Coding Interactive Behavior Manual; CoP – Copeptin; CORT – Cortisone; CRIES – Children's Revised Impact of Event Scale; DEX – Dexamethasone; DHEA – dehydroepiandrosterone; DSM-IV-TR – Diagnostic and Statistical Manual of Mental disorders IV edition, Text Revision; EA – Early Adolescent; ELSI – Early Life Stress Inventory; ERIWQ – Effort-Reward Imbalance at Work Questionnaire; GSE – Generalized Self-Efficacy Scale; HCC – Hair Cortisol Concentration; HI – Human Insecurity; HS – High Stress; IPV – Intimate Partner Violence; ISR – Israel; JOR – Jordan; LA – Late Adolescence; LC – Late Childhood; LEB – Lebanon; LOT-R – Life Orientation Test - Revised; MBI – Maslach Burnout Inventory; Mage – Mean age; MET – Metyrapone; PCL-5 – Post-Traumatic Stress Checklist; PDS – Post-Traumatic Diagnostic Scale; PROVE battery - PROtective and Vulnerable factors battEry; PRS – Polygenic Risk Score; PTSD – Post Traumatic Stress Disorder; PSS – Perceived Stress Scale; RE – Resilient Exposed; SAA – Salivary Alpha Amylase; SAU – Saudi Arabia; SC – Salivary Cortisol; SCARED – Screen for Child Anxiety Related Emotional Disorder, mother-report and child's self-report; SDHEA – sulfated dehydroepiandrosterone; SKR – South Korea; STAI-Y – State-Trait Anxiety Scale for adults; TSST – Trier Social Stress Test; UK – United Kingdom; USA – United States of America; WHO-5-WBI – WHO 5 Well Being Index; y.o. – years old.; ZEN – high control over emotions.

Paralleling clinical data on human cortisol and HPA axis response, compelling evidence in rodents has reported a key role for corticosterone, the main corticosteroid hormone in rodents (Smith and Vale, 2006), as a biomarker of stress vulnerability. Elevated corticosterone levels were found to be associated with the development of depressive-like phenotype in rodents (Kott et al., 2016; Dwivedi et al., 2015; Malkesman et al., 2006; Overstreet et al., 2005), while PTSD-like animal models are generally characterized by low plasma corticosterone levels (Verbitsky et al., 2020; Borghans and Homberg, 2015), such that low basal corticosterone pulse amplitude has been identified as a predictive variable for PTSD-like susceptibility (Danan et al., 2018). These decreased corticosterone levels induce a blunted glucocorticoid responsiveness, which in turn is thought to be responsible for PTSD-related alterations, such as fear memory dysfunctions and REM sleep disturbances (Reznikov et al., 2015; Monari et al., 2023).

Different single nucleotide polymorphisms (SNPs) of corticotropin-releasing hormone receptors type 1 and 2 (CRHR1 and CRHR2, respectively) have been reported to affect the risk and severity of PTSD in humans (White et al., 2013; Wolf et al., 2013). Rodent studies have identified a key role for CRHR2 receptor overexpression in the bed nucleus of stria terminalis to reduce PTSD-like alterations in susceptible animals (Elharrar et al., 2013). Moreover, mice with CRH overexpression exposed to an early-life stressor displayed susceptibility to develop PTSD-like behavioral alterations later in life (Toth et al., 2016).

Alterations of the glucocorticoid signaling pathway have been associated with susceptibility to developing a PTSD-like phenotype. Transcriptomic analyses of blood and brain samples in rodents have revealed an important role for GR signaling in shaping the interindividual variability in stress and trauma responsivity (Daskalakis et al., 2014). It has been found that single prolonged stress (SPS), a PTSD-like experimental model consisting of exposure to a psychological (i.e. restraint), physical (i.e. forced swim stress) and pharmacological (i.e. gas anesthesia) stressor in one prolonged session, induced PTSD-like behavioral alterations linked with increased hippocampal GR expression and activation in rats (Liberzon et al., 1999; Kohda et al., 2007), which may represent one of the mechanisms underlying the enhanced HPA axis feedback observed in PTSD patients. Due to its ability to reduce GR activation through the formation of a GR-FKBP5 complex (Wochnik et al., 2005), the *FKBP5* gene has been identified as a potential candidate biomarker for stress related disorders (Zannas et al., 2016). This is supported by evidence reporting that mice overexpressing *FKBP5* gene exposed to maternal separation showed depressive-like behaviors later in life (Criado-Marrero et al., 2020), and that aged animals presenting elevated FKBP51 levels show impaired resiliency to depressive-like behaviors, a phenotype that is abrogated in FKBP5^−/−^ mice (Sabbagh et al., 2014). Pituitary adenylate cyclase-activating polypeptide (PACAP) regulates stress adaptation and has been related to the development of stress-related disorders in both humans and rodents (Pituitary adenylate cyclase activating polypeptide), thus representing a potential candidate biomarker of stress resilience. Previous evidence has demonstrated that chronic stress enhances brain expression of PACAP and PACAP receptor type 1 (PAC1) and that mice treated with a central infusion of PACAP display an anxious-like phenotype (Hammack et al., 2014; Hammack et al., 2009). Conversely, PAC1-deficient mice present reduced anxious-like behaviors (Otto et al., 2001). Lastly, recent evidence has also identified MR as a potential candidate for stress resilience (ter Heegde et al., 2015). Chronic stress exposure reduces hippocampal MR expression (López et al., 1998) and induces vulnerability to depressive-like behaviors in male mice (Schmidt et al., 2010). Moreover, mice with forebrain MR overexpression exhibited increased memory performances and reduced anxious-like phenotype (Lai et al., 2007).

### 4.2 Endocannabinoid system

The central role exerted by the endocannabinoid system in mediating stress effects at the molecular, circuit, and behavioral levels has been extensively demonstrated (Morena et al., 2016). Converging preclinical and clinical evidence points at the endocannabinoid system components as potential promising biomarkers for stress-related disorders (recently reviewed in Yirmiya et al., 2018; Smeeth et al., 2023; Li et al., 2019; Barry et al., 2021).

Genetic variations of different endocannabinoid components have been reported to be involved in the vulnerability to develop stress- and anxiety-related disorders in humans. For instance, specific genetic variations in the *CNR1* (gene coding the Cannabinoid type 1 Receptor, CB1) rs7766029 polymorphism have been associated with the development of depression and anxiety (Gonda et al., 2019). A *FAAH* 385C→A loss-of-function mutation encodes a fatty acid amide hydrolase (FAAH), the enzyme responsible for the endocannabinoid anandamide (AEA) degradation, that is degraded more rapidly, thus producing elevated peripheral AEA levels (Dincheva et al., 2015; Mayo et al., 2020; Sipe et al., 2002). Subjects homozygous for the A-allele present enhanced fear extinction, which is related to elevated peripheral AEA levels (Spohrs et al., 2021; Ney et al., 2021), and are resistant to stress-induced decreases in blood AEA levels (Mayo et al., 2020), thus suggesting that AEA buffers against the negative behavioral consequences of stress (Morena and Campolongo, 2014). Subjects with PTSD and comorbid alcohol use disorder who carry the variant A-allele also have higher peripheral AEA levels (Spagnolo et al., 2016) and show greater improvements on the PTSD symptom of hyperarousal. Accordingly, another study reported that PTSD was associated with reduced circulating AEA levels accompanied by an upregulation of CB1 receptors within the amygdala-hippocampal-cortico-striatal neural circuit, compared with a trauma and healthy control group (Neumeister et al., 2013). However, exposure to repetitive childhood trauma or genetically conferred hyper-reactivity of the HPA axis in patients carrying the A allele increases the vulnerability to developing anxiety and depression (Lazary et al., 2016; Demers et al., 2016). Animal studies parallel this evidence in humans, showing that increased AEA levels promote fear extinction and reduce anxiety (Morena et al., 2016; Gunduz-Cinar et al., 2013a; Gunduz-Cinar et al., 2013b; A cortico; Emotional arousal state influences the; Colangeli et al., 2020). Anxiolytic effects induced by increased endocannabinoid 2-arachidonoyl glycerol (2-AG) tone have also been reported in different animal models (Morena et al., 2016; Bosch-Bouju et al., 2016; Sciolino et al., 2010; Bedse et al., 2017; Lim et al., 2016; Bluett et al., 2017). Enhancing AEA levels through the administration of the FAAH inhibitor URB597 ameliorates fear extinction profile and sociability (Morena et al., 2018) and reduces hyperarousal as well as anxious-like behavior in rats exposed to PTSD paradigms (Fidelman et al., 2018). Conversely, low AEA levels within several stress-sensitive brain areas were reported in rats previously exposed to an animal model of depression (Hill et al., 2008). Interestingly, if brain (in rodents) and peripheral (in both rodents and humans) AEA levels are augmented immediately after a traumatic event, it enhances fear memory consolidation (Hill et al., 2008) and increases the risk to later develop PTSD (deRoon-Cassini et al., 2022). Accordingly to the evidence mentioned above that increased AEA and 2-AG signaling buffers against the negative consequences of stress exposure, pharmacological CB1 receptor blockade or their genetic deletion increase freezing in mice subjected to a PTSD paradigm (Bowers and Ressler, 2015) and impairs extinction of fear (Marsicano et al., 2002), whereas a pharmacologic CB1 receptor stimulation significantly reduces freezing behavior in rats exposed to chronic-mild-unpredictable stress (Reich et al., 2013). CB1 receptors knockdown mice exposed to chronic unpredictable stress have been found to be more susceptible to the development of depressive-like behaviors and anhedonia (Martin et al., 2002). Moreover, rats exposed to a PTSD-like model showed lower CB1 receptor expression levels compared to controls (Xing et al., 2011).

Altogether, these data provide solid evidence that endocannabinoid system components may serve as reliable biomarkers for stress resilience (Hill and Lee, 2016; Worley et al., 2018). Table 2 summarizes the main results of the selection of studies reporting on clinical models for biomarkers focusing on the endocannabinoid system.

**Table 2:** Selection of paper reporting on clinical models of biomarkers involving the endocannabinoid system.

Author, year	Study design	Sample size	Sample characteristics	Diagnostic category or clinical correlate	Main outcomes reported	Main results	Exclusion criteria	Country
Demers et al., 2016	Cross-sectional study	N = 661 -Mean age 19.6 ± 1.2, female 55,6%	-Bipolar disorder - GAD - PD - AGO - Alcohol abuse and dependence - Cannabis abuse and dependence - OCD - Social Anxiety	MINI	FAAH rs324420 and CRHR1 rs110402 polymorphisms effects on amygdala function and anxiety disorder diagnosis.	- Blunted basolateral amygdala habituation with a genetic background associated with high AEA and CRHR1 signaling - Significant association between left basolateral amygdala habituation and risk for anxiety disorders	- Conditions affecting cerebral blood flow (e.g., arterial hypertension) - Medical disorders (e.g., cancer, stroke, insulin-dependent diabetes, chronic kidney disease, liver disease) - Lifetime psychotic diagnoses - Psychotropic, glucocorticoid or hypolipemic medications - Contraindications to MRI scanning	USA
deRoon-Cassini et al., 2022	Prospective cohort study	- n = 278 baseline - n = 170 follow-up assessment	- Mean age 42.8 ± 16.5 y.o., 30% female-	PTSD - PCL-5 - CAPS Biomarkers: serum AEA, 2-AG and plasma cortisol - Genotypes FAAH (rs324420), CNR1 (rs1049353, rs806371, rs2180619)	Association of selected biomarkers with a longitudinal evaluation of PTSD symptoms development following trauma among hospitalized subjects receiving care	- Serum 2-AG and AEA levels correlated significantly with PTSD symptoms among subjects belonging to ethnic minorities - A/A genotype of rs324420 was associated with higher PTSD symptoms severity but only in Afro-American subjects	- Non-English speaking - Greater than mild traumatic brain injury - Being detained by law enforcement in the hospital - Intentional self-injury	USA
Golia et al., 2019	Cross-sectional study	1346 UK 923 HUN	NewMoon study cohort	- BSI - LTE Biomarkers: genotypes for CNR1 receptor variant rs7766029, GABRA gene variant rs3219151	Evaluate the possible association of BSI scores, LTE with tested genotypes	- Significant interaction of CNR1rs7766029 and financial-related events on BSI-defined anxiety and expression scores - GABRA6 rs3219151 interaction with social-network-related life-events on BSI anxiety, and with an illness-personal problem related on BSI depression score	N/A	HUN, UK
Lazary et al., 2016	Cross-sectional study	858	- Mean age 31.27 ± 10.5 y.o., female 69.8% - Volunteers	- ZSDS - BSI-DEP - BSI-ANX - STAI-S - STAI-T - CHA	Evaluation of the interaction between FAAH genetic variations, childhood adversity and their association with anxiety and depression	- Allele A carriers of the FAAH C385A polymorphism presented higher CHA scores and higher anxiety and depression scores as compared with CC carriers	N/A	HUN
Mayo et al., 2020	Experimental study	75	- Mean age 24.4 ± 0.4; Female 52%	Biomarkers: blood AEA, 2-AG, OEA, PEA, cortisol	Assessing differences in fear-conditioning response depending on the FAAH genotype in the context of an experimental stress exposure - a 2-day fear conditioning paradigm, stress task (MAST), affective image task (IAPS) - Evaluate differences in selected biomarkers	- FAAH 385A allele appeared associated with higher AEA levels, facilitated fear extinction, higher extinction recall, was protected from the emotional consequences of stress	N/A	SWE
Neumeister et al., 2013	Cross-sectional study	60	− 25 PTSD (mean age 32 ± 9.9 y.o., female 56%) − 12 TC (mean age 29 ± 7.9 y.o., female 41.7%) − 23HC (32.1 ± 8.5 y.o., 52.2 % female)	PTSD - CAPS Biomarkers: cortisol, AEA, PEA, OEA, 2-AG - Brain-wide [11C]OMAR VT value (indicating CB1 receptor availability)	Association between selected biomarkers and PTSD symptoms severity	The PTSD group presented elevated brain-wide [11C]MAR VT values, with a negative correlation of [11C]MAR VT values with AEA levels	- Known medical or neurological conditions - Substance abuse within 12 months prior enrollment - Lifetime history of substance dependence - Head injury with loss of consciousness history	USA
Sipe et al., 2002	Cross-sectional study	- Problem drug use/drug disorder (n = 80) - Negative self-report drug use (n = 1737)	Mixed population of self-reported illicit substance users and subjects with diagnosed psychiatric conditions (autism, bipolar spectrum disorders, schizophrenia)	- Drug and alcohol use - Schizophrenia, Bipolar disorder, autism diagnoses	Evaluate the association FAAH 385C3A missense mutation with problem drug and alcohol abuse	Significantly different distribution for FAAH 385A allele distribution between street drug use and problem drug or alcohol use as compared with controls	N/A	USA
Spagnolo et al., 2016	Cross-sectional study	49	- Mean age 40 ± 7.9, female 49%	PTSD and comorbid alcohol dependence - ASI - NEOPI-R - CTQ - SUDS - STAI-S -PSSI Biomarkers: - serum AEA, OEA, PEA, 2-AG	Association of FAAH C385A genotype, AEA, stress, anxiety and PTSD symptoms	FAAH385A allele carriers presented higher AEA levels and lower subjective anxiety levels, with a significant impact on the arousal domain of PTSD symptoms	- Unable to provide consent - Advanced liver disease	USA
Spohrs et al., 2021	Experimental study	51	- Male 100% - Mean age 22.8 ± 3 y.o.	Evaluating the association of AEA levels with fear extinction learning	- Brain activation during fear extinction learning as evaluated through fMRI Biomarkers plasma AEA	- Higher AEA levels associated with stronger fear extinction learning - AEA changes correlated with neural activation patterns	- Medical and psychiatric conditions - Substance use	GER

Abbreviations: 2-AG – 2-arachidonoylglycerol; AEA – Anandamine; ASI - Addiction Severity Index; BSI – Brief Symptoms Index; BSI-DEP, BSI-ANX – Brief Symptoms Index Anxiety and Depression subscales; CHA – Childhood Trauma Questionnaire; CTQ – Childhood Trauma Questionnaire; fMRI – Functional Magnetic Resonance Imaging; GER – Germany; HC – Healthy Controls; HUN – Hungary; IAPS – International Affective Picture System; LTE – List of Threatening experience; MAST – Maastricht Acute Stress Test; NEOPI-R - NEO Personality Inventory-Revised; OEA – oleoylethanolamide; PCL-5 – PTSD Checklist −5; PEA – palmitoylethanolamide; PSSI - Posttraumatic Stress Disorder Symptom Severity Index; SUDS – Subjective Units of Distress Scale; STAI-S – Spielberger State Trait Anxiety Inventory; STAI-S, STAI-T - State Trait Anxiety Inventory; SWE – Sweden; TC – Trauma Exposure Controls; UK – United Kingdom; ZSDS – Zung Self-Rating Depression Scale.

### 4.3 Endogenous opioid system

It is well established that opioid signaling is sensitive to stress exposure (Lutz et al., 2018; Nakamoto et al., 2020; Torres-Berrio and Nava-Mesa, 2019). For their analgesic effects, enkephalins have been proposed as targets to promote stress resilience (Ryan and Ryznar, 2022; Henry et al., 2017). Preproenkephalin-knockout rodents show increased immobility in an auditory fear conditioning task (Ragnauth et al.), hyperarousal responses as well as reduced sociability (Bilkei-Gorzo et al., 2004), anxious- and depressive-like behaviors (Kung et al., 2010), while a lower level of enkephalin expression has been associated with anhedonia (Poulin et al., 2014). Additionally, converging evidence has reported that stress exposure alters enkephalin signaling (for a comprehensive review of all these effects in rodents see 115). These data indicate a potential role of enkephalins in promoting stress resilience in rodents. Rats subjected to chronic social defeat stress exhibit decreased mRNA enkephalin levels in the basolateral amygdala (BLA, 120), and BLA enkephalin knockdown in mice induces anxious-like alterations similar to those observed after stress exposure (Bérube et al., 2014). Further studies have reported that mice carrying the mu-opioid receptor type 1 (OPRM1) A118G polymorphism or treated with a delta-opioid receptor (DOR) agonist exhibit resilience toward the development of negative emotional outcome of social defeat stress (Briand et al., 2015; Henry et al., 2018). Positive effects induced by the stimulation of the opioid system have also been demonstrated in animal models of PTSD treated with morphine (RaiseAbdullahi et al., 2019).

### 4.4 Immune system

Despite the evident importance of the immune system for the species' survival and the evolutionary role it has, a failure to reinstate physiological homeostasis may be recognized as one of the many elements underlying the development and persistence of numerous pathological conditions (Daskalakis et al., 2016). In recent years, the connection between the immune system and the brain has been extensively explored (Ader and Cohen, 1975; Besedovsky et al., 1983; Bullmore, 2018; Milaneschi et al.). Immune dysregulation has been linked with the vulnerability and onset of various psychiatric conditions, including schizophrenia, bipolar disorder, and autism spectrum disorder (Benedetti et al., 2020; Croonenberghs et al., 2002; Khandaker and Dantzer, 2016), and special attention has been paid to the role of immune alterations in major depressive disorder (Dantzer, 2012; Miller and Raison, 2016). In the preclinical models, early life stress paradigms have been associated with derangements in inhibiting immune pathways in the developing hippocampus (Wei et al., 2012) and in enhanced Toll-like receptor-dependent cytokine secretion (Powell et al., 2009). A 2014 report comprising 2208 participants evaluated prospectively the C-reactive protein levels of US marines before and after war deployment. At the 3-months follow-up, adjusting for the severity of trauma exposure, low-grade inflammation was associated with a greater risk of developing PTSD (Eraly et al., 2014). Approximately 30% of depressed individuals exhibit high levels of inflammatory markers in the blood (Miller and Raison, 2016; Osimo et al., 2019; Raison et al., 2006, 2013a), including Interleukin (IL)-1β, IL-6, Tumor necrosis factor (TNF)-α, and C-reactive protein (CRP) (Benros et al., 2013a; Dickens and Creed, 2001). A recent meta-analysis encompassing 30 studies reveals that 27% of individuals with depression experience chronic low-grade inflammation (CRP >3 mg/L), while the majority (58%) of patients show CRP levels exceeding 1 mg/L (Osimo et al., 2019). In addition, TNFα, predict the onset of major depressive disorder over the subsequent months or years in otherwise healthy individuals (Baune et al., 2012a; Khandaker et al., 2014a; Lamers et al., 2019a; Milaneschi et al., 2009a; Miller et al., 2019a) and intravenous administration of pro-inflammatory agents triggers the appearance of depressive symptoms (Capuron and Miller, 2011; Eggermont et al., 2008; Engler et al., 2017a; Friebe et al., 2010; Lasselin et al., 2020; Madeeh Hashmi et al., 2013).

The brain-immune crosstalk in major depressive disorder is corroborated by recent studies indicating that immune overactivation can diminish the effectiveness of antidepressant drugs (Benedetti et al., 2021a; Carvalho et al., 2013a; Colpo et al., 2018; Haroon et al., 2018; Pariante, 2017; Strawbridge et al., 2015), suggesting the role of inflammatory processes in antidepressant action. Patients with elevated expression levels of immune activation-associated genes, such as IL-6, TNF-α, and IL-1β, in their blood show a significantly reduced response to various classes of antidepressants like selective serotonin reuptake inhibitors (SSRIs) or tricyclic antidepressants (Arteaga-Henriquez et al., 2019; Cattaneo et al., 2016; Eller et al., 2008a; Lanquillon et al., 2000a; Tuglu et al., 2003a) Conversely, a meta-analysis has shown that antidepressant treatment significantly reduces TNF-α levels in responders only (Liu et al., 2020).

Numerous molecular and cellular mechanisms have been postulated to link alterations in the immune system and the onset of major depressive disorder, as well as the effectiveness of therapeutic interventions. One of the extensively researched pathways in this context is the kynurenine pathway, implicated in neuropsychiatric disorders and proposed as a crucial link between inflammation and depression (Dantzer et al., 2008; Haroon et al., 2020a; Vancassel et al., 2018). Supporting this concept, the activation of the kynurenine pathway has been demonstrated to correlate with the severity of neuropsychiatric symptoms in clinical populations (Parrott et al., 2016; Raison et al., 2010a; Savitz et al., 2015a, 2015b). Notably, particular emphasis has been placed on indoleamine 2,3-dioxygenase (IDO), given its association with elevated levels of depression-like phenotypic markers in preclinical models (Andre et al., 2014; Dinel et al., 2014; O’Connor et al., 2009a; O’ Connor et al., 2009b). Given the critical role of immune activation in the vulnerability to psychopathology, particularly in major depressive disorder (Milaneschi et al., 2020; Pariante, 2017; Blume et al., 2011; Lamers et al.a; Maes et al., 1995a), immune activation has been proposed as a key player in fostering resilience against psychiatric disorders. Metabolic dysregulation represents a further potential mechanism when exploring the nexus between immune activation and major depressive disorder. This interplay manifests in a substantial proportion of depressed patients, ranging from 15% to 29% (Milaneschi et al., 2020). For instance, pathways involving white adipose tissue, particularly in the abdominal region, emerge as active endocrine organs generating inflammatory cytokines and hormones (e.g., leptin) (Chait and den Hartigh, 2020). Consequently, they become major contributors to pathogenic immune-metabolic responses, impacting both the central nervous system and the rest of the body (Chait and den Hartigh, 2020). A final example of potential mechanisms by which immune dysfunction may affect mental health concerns the dysregulated balance between innate and adaptive immunity and between the pro-inflammatory and anti-inflammatory/regulatory branches of the immune system. Indeed, accumulating evidence suggests that cell-mediated immunity actively contributes to the pathogenesis of major depressive disorder (Beumer et al., 2012; Miller, 2010; Toben and Baune, 2015). Conditions that trigger a pro-inflammatory profile are proposed as mechanisms potentially undermining resilience, thereby contributing to the onset of mental illness. Accordingly, anti-inflammatory drugs have been proposed and tested as effective treatments in psychiatry, and several drugs dampening immune overactivation are currently under investigation for treating depression (Fourrier et al., 2018; Kohler et al., 2014; Kopschina Feltes et al., 2017; Muller, 2019; Rosenblat et al., 2014; Tyring et al., 2006). NSAIDs, which inhibit Cyclooxygenases (COXs) and reduce inflammation, are being studied as standalone treatments or adjuncts to standard antidepressants (Baune, 2016). Celecoxib, a COX-2 inhibitor, has shown effectiveness when combined with traditional antidepressants or used as monotherapy (Kohler et al., 2014, 2016; Abbasi et al., 2012a; Akhondzadeh et al., 2009; Muller et al., 2006; Na et al., 2014). Non-selective COX-2 inhibitors, like acetylsalicylic acid, have also demonstrated antidepressant properties (Berk et al., 2013; Kessing et al., 2019). Minocycline, an antibiotic with anti-inflammatory and neuroprotective effects, shows promise as an adjunctive treatment for depression (Dean et al., 2012; Miyaoka et al., 2012; Nettis et al., 2021; Pae et al., 2008; Soczynska et al., 2012). A recent review of meta-analyses appears to confirm the impression of a promising role for several medications in MDD, especially for celecoxib, despite the significant heterogeneity in terms of patient selection, inflammatory status, disease duration, clinical outcome scale, and treatment regimen for the selected papers (Simon et al., 2023; Simon, 2023). Table 3 summarizes the main findings for the selection of papers reporting on inflammatory biomarkers in clinical models.

**Table 3:** Selection of studies reporting on clinical models for inflammatory biomarkers.

Author, year	Study design	Sample size	Sample characteristics	Diagnostic category or clinical correlate	Main outcomes reported	Main results	Exclusion criteria	Country
Abbasi et al., 2012b	RCT	37	- Sertraline + celecoxib = 35.1 ± 8.0, female 35% - Sertraline + placebo = 34.2 ± 6.9, female 30%	MDD - HDRS Biomarkers: serum IL-6	Association of IL-6 level change with depressive symptoms from baseline during a 6-week course of either sertraline + celecoxib or sertraline + placebo treatment	- The sertraline + celecoxib group presented greater IL-6 reductions compared to the control group - Reduction of HDRS score correlated with reduction of IL-6 at week 6	- Other Axis I or II diagnoses - Recent antidepressant use - High suicide risk - Pregnancy or lactation - recent ECT - Substance use or dependence	IRN
Baune et al., 2012b	Prospective cohort study	1037	- Female 55.2 - <75 y.o. (n = 268) − 75-79 (n = 358) - ≥80 (n = 411)	- GDS - GAS - MMSE	- Association of mood and biomarkers at:1) baseline; 2) 2-year follow-up, 3) remitted depression at baseline, 4) first onset of depression at follow-up - Biomarkers (IL-) -1b, −6, 8, −10, −12p70, sVCAM-1, PAI-1, SAA, TNF-a and CRP	Association between Il-6 and depressive symptoms at baseline, Il-8 associated with first onset mild to moderate depression and with depressive symptoms at baseline and follow-up; PAI-1 associated with remitted depression	- Dementia - Developmental disabilities - Psychotic symptoms - Schizophrenia or bipolar disorder - Multiple sclerosis - Motor neuron disease - Progressive malignancy - Inadequate English level	AUS
Benedetti et al., 2021b	Observational study	108	MDD inpatient, with ongoing antidepressant therapy - Female 62% - Age: SSRI group 50 ± 10.75, SNRI group 52.13 ± 8.8	Association between MDD treatment response and selected biomarker levels	- HDRS Biomarkers: IL−1β - IL-1rα - IL-2 - IL-4 - IL-5 - IL-6 - IL-7 - IL-8 - IL-9 - IL-10 - IL-12(p70) -IL-13 - IL-15 - IL-16 - IL-17 - IFN-γ - TNFα - MCP1/CCL2 - MIP-1α/CCL3 - MIP-1β/CCL4 - RANTES/CCL5 - CCL11 - IP-10/CXCL10-FGF - G-CSF - GM-CSF - PDGF-B - VEGF	Higher baseline levels of IL−1β and TNFα were associated with lower response rates to selected pharmacological therapy	- Axis I diagnoses - Pregnancy - Medical co-morbidities - Recent LAI (<3 months) - History of alcohol or illicit substance abuse - Infectious or inflammatory disease - Somatic disorders with possible mood impact	ITA
Benrose et al., 2013b	Retrospective cohort study	3.56 million people (78 million person-years follow-up)	N/A	Mood disorder diagnosis received in a hospital, outpatient clinic or emergency department	Risk of lifetime diagnosis of mood disorder association with autoimmune or infectious disorders	Any history of autoimmune or infectious disorder increases the risk of receiving a subsequent mood disorder diagnosis	N/A	DEN
Carvalho et al., 2013b	Experimental study	40	− 19 TRD − 21 HC - Female 72.5%	TRD	- HDRS - BDI - BAI - BSI - BHS - RLCQ Biomarkers: plasma cortisol, serum cytokines (VEGF, MCP-1, IL-6, IL-4, IL-10)	Depression symptoms showed higher levels of cortisol, IL-6 and Il-10 and lower levels of IL-4, VEGF compared to HC - Relatively lower MCP-1 and VEGF in refractory inpatients	- Hypersensitivy to corticosteroid - heavy smokers - Pregnancy or lactation - Significant physical illness - Drug therapy for immune or endocrine functions	UK
Cattaneo et al., 2016	Secondary analysis, GENDEP cohort	74	GENDEP cohort	MDD – drug-free at enrollment - mean MADRS 29.5 ± 3.9	Assessing the predictive response of mRNA MIF and IL-1β for antidepressant treatment response (baseline through 12-weeks MADRS levels)	Absolute mRNA measures accurately predicted response probability on an individual basis in a GENDEP population and an independent replication sample	- Antipsychotics or mood stabilizers - Comorbidity with Axis I or II - Substance abuse - Head injury - Severe medical illness	Multiple European centres
Croonenberghs et al., 2002	Cross-sectional study	26	- Male 12–18 y.o. − 13 ASD, 13 HC	ASD	Comparison of whole blood IL-6, IL-10, IL-1RA, INF- γ, TNF-α and serum IL-2R, IL-1RA and IL-6 levels between ASD and HC	Increased levels of INF- γ and IL-1RA and trend for increased IL-6 and TNF-α in ASD	HC: -negative past or present psychiatric family history -Free of medication and substance abuse for one month ASD and HC: Neurological, inflammation, endocrine or other significant chronic diseases - Active seizure disorder - Chromosomal disorders - Drugs with potential to interact with endocrine/immune system	BLG
Engler et al., 2017b	Experimental study	18	- Male 100% - Healthy volunteers (10 endotoxin group; 8 placebo group) − 27.8 ± 1.2 y.o.	- HADS - Biomarkers: TNF-α, IL-6, IL-10 and IL-1β (plasma and CSF)	Assessing the association between endotoxin exposure and anxiety and depression symptoms	- Endotoxin administration associated with higher CSF IL-6 levels - IL-6 levels correlated with depression symptoms	- Current physical or psychiatric disorder - Smoking - Substance use disorder - BMI <18 or ≥ 29 - Current pharmacological therapy - screening positive for HADS	GER
Eller et al., 2008b	Experimental study	145	− 100 MDD − 45 HC - Female 65% − 32.1 ± 11.9 y.o.	MADRS Biomarkers - IL-8 - TNF-α - sIL-2R	Assessing selected cytokine levels (assessed at baseline, 4th week and through 12th week of treatment) and escitalopram response	Higher TNF-α levels at baseline was associated with higher levels non-response to escitalopram treatment	- Acute infections - Neurological or immunological disorders - Substance use disorder - Bipolar or panic disorder	EST
Eraly et al., 2014	Prospective cohort study	2215	- Male 100% Mean age 22.8 y.o.	PTSD - CAPS - BDI - BAI	Assessing the association PTSD symptoms development 3-months post-deployment (T1) and baseline CRP levels	Baseline CRP significantly associated with T1 CAPS scores	N/A	USA
Haroon et al., 2018	Observational study	98	− 21-65 y.o. - Female 66.3%	TRD (MGHATRQ)	- HDRS Biomarkers: IL-1β - IL-6 - IL-6sr - IL-10 - TNF-α - sTNF-αR2	Increased inflammatory markers levels appeared associated with greater number of failed trails (especially, TNF, s-TNF-r2 and IL-6	- Current suicidal ideation - Psychiatric diseases other than depression - Autoimmune or inflammatory disorders - Recent acute or chronic infection (bacterial, fungal or viral) - History of cancer - hematologic, renal, hepatic, endocrine or neurologic disease - Diabetes or glycosuria - Pregnancy or lactation	USA
Haroon et al., 2020b	Cross-sectional study	72	- Female 67% - mean age 39.38 (CI 95%: 42.15–36.6)	HDRS IDS-SR MGHATQR - Biomarkers Plasma: - hs CRP - KYN - IL-1β - IL-1RA - MCP1/CCL2 - TNF2R - IL-6 - IL-6SR - TRP - KYN - KYNA - 3HKYN - AA - 3HAA - QA CSF: - CRP - TRP - KYN - KYNA - 3HKYN - AA - 3HAA	Evaluate the association between depressive symptoms and selected plasma biomarkers levels	KYN pathway metabolites in CSF and plasma levels appear associated with plasma TNF levels - High TNF.KYN/TRP subjects feature greater depression, anhedonia and treatment non-response	- Autoimmune conditions - HBV, HCV, HIV infections - Oral glucocorticoid in the 6 months prior to enrollment - regular NSAID treatment - Current suicidal ideation - History of psychotic disorders - Substance abuse past six months - No mood stabilizer, antipsychotic, or benzodiazepines in the four weeks prior to enrollment (8 weeks for fluoxetine)	USA
Khandaker et al., 2014b	Prospective cohort study	4585	ALSPAC birth cohort –	- Psychosis and depression diagnosis risk - CIS-R - MFQ - Semistructured interview For psychotic experience	From age seven, patients were assessed through age 18 for depression (n = 2453) and psychosis (n = 2528) risk	Higher Il-6 was associated with an increased risk of depression and psychosis at follow-up	Infection at the time of blood collection or in the preceding week	UK
Lamers et al., 2019	Prospective cohort study	- baseline (n = 2416) - 2-years follow-up (n = 1925) - 6-years follow-up (n = 1924)	- Baseline 41.9 ± 12.9 y.o. - Female 66.3%	Adult subjects with and without MDD	Evaluate the association between IL-6 and CRP with	- Higher Il-6 levels was associated with higher chronicity levels of depression	- BD, OCD, psychotic disorders, or severe addiction disorders - Inadequate Dutch language level	NET
Lamers et al., 2019b	Prospective cohort study	2981baseline − 2241 2-year follow-up (T1) − 1955 6-year follow-up (T2) NESDA cohort	- Mean age 41.9 ± 13.1 y.o. - Female 66.4% (baseline)	MDD, or anxiety disorders (GAD, PD, AGO, SP) - IDS - BAI - CTQ Biomarkers Plasma: IL-6. FG. HDL. TRYG	- Depression profiles and immune-metabolic indices - Inflammation index, metabolic syndrome index, combined inflammation-metabolic	- Immuno-metabolic indices positively correlated with atypical energy-related symptoms - Melancholic symptoms negatively associated with metabolic syndrome index	- Severe psychiatric disorder - Inadequate Dutch proficiency level	NET
Lanquillon et al., 2000b	Experimental study	24	- Female 62.5% − 53.5 ± 15.2 y.o.	MDD	Association of HDRS and MADRS scores after 6-weeks of amitriptyline treatment with selected biomarkers levels - Biomarkers: CRP, IL-6, TNF-α	- Higher CRP levels in MDD patients (responders and non-responders) compared to HC - TNF-α levels decreased only among responders - Higher pretreatment IL-6 levels in the non-responder group	- Axis I or II co-morbidity - Pregnancy - Acute or chronic infection - Acute physical illness, surgery, myocardial or cerebral infarction in the three months preceding enrollment	GER
Maes et al., 1995b	Cross-sectional study	99	− 68 MDD - Minor depression 41.2 ± 11.9; Simple major depression 42.1 ± 13.4; Melancholia 52.8 ± 13.3 − 22 HC (41.2 ± 13.9)	HDRS Biomarkers: - serum IL-1RA - Urinary cortisol and post-DEX cortisol	Comparison of Serum IL-1RA, urinary cortisol post-DEX between groups	MDD subjects presented higher IL-1RA - Positive correlation between IL-1RA and symptom severity - No association between HPA axis activity and serum IL-1RA	- Other axis I diagnoses - Treated with MAOIs, lithium, high-dose neuroleptics, barbiturates, fluoxetine - ECT in the year prior to enrollment - Abnormal physical, blood, and urine analyses - Chronic endocrine or immune disorders	BLG
Milaneschi et al., 2009b	Prospective cohort study	991	InCHIANTI study cohort	- Adults ≥65 y.o. - CES-D - Biomarkers: TNF-α - IL-6 - IL-6 receptor - IL18 - CRP - IL-1β	Evaluate the association of depressive symptoms and selected biomarkers at baseline and at 3- and 6- years follow-up (T1 and T2, respectively- depression = CES-D ≥20)	Higher IL-1RA at baseline is associated with higher risk for depressive symptoms at T2	Lack of clinical or biomarker assessment	ITA
Miller et al., 2019b	Prospective cohort study	117	- Women, singleton gestation scheduled for cesarean section	IDS≥18 Biomarkers: IL-1β - INF- α - TNF- α - MCP-1 - IL-6 - IL-8 - IL-10 - IL12p70 –IL-17A - IL-18 - IL-23 - IL-33	Assess the association between perinatal depression and selected CSF biomarkers	No association between plasma cytokines and depressive symptoms - Positive association for CSF IL-1b, IL-23, IL-33 and perinatal depression	- Diabetes - Preeclampsia - ≤18 y.o. - Anti-inflammatory medications - HIV - Fetal anomalies	USA
Nettis et al., 2021	RCT	44	− 25-60 y.o. − 22 minocycline augmentation, female 55.6% − 22 placebo augmentation, female 57.1% - HDRS ≥14	MDD - HDRS - BDI - CTQ - CGI-S - BLE - PSS - SHAPS - STAI Biomarkers: serum -hsCRP - IFN-γ - IL-1β - IL-2 - IL-4 - IL-6 - IL-8 - IL-10 - IL-12p70 - IL-13 - TNF-α	- Assess efficacy of Minocycline augmentation to standard treatment after failure to respond to standard treatment - Assess the association of selected biomarkers with symptom variation	- IFN-γ was significantly reduced after minocycline augmentation but not placebo - Minocycline appeared efficacious in MDD subjects with CRP≥3 mg/L	- Active suicidal ideation - Bipolar disorder, OCD, eating disorder, PTSD - Warfarin therapy - Tetracycline therapy two months prior enrollment - Acute infection - Autoimmune or inflammatory conditions - Hepatic or renal failure - Other psychotropics prior to enrollment	UK
Raison et al., 2010a	Experimental study	27	- HCV patients (n = 16 undergoing 12-weeks INF/RIBn treatment; n = 11 waiting treatment)	MADRS Biomarkers - Plasma: KYN, QUIN - CSF: KYN, QUIN, KA	Association of TRP metabolite pathway in plasma and CSF with depressive symptoms in subjects receiving INF/RIB treatment for HCV	- INF-RIB treated patients presented higher QA, KYN and KYNA in CSF - Plasma KYN highly correlated with QA - TRP, KYN and KYN/TRP	- Unstable cardiovascular, hematologic, renal or neurologic disease - HIV infection - Liver disease other than HCV - Bipolar disorder - MDD - Substance use disorder 6-months before enrollment - Cognitive impairment	USA
Raison et al., 2013b	RCT	60	- Female 66% (both grops) − 42.5 ± 8.2 y.o. (Infliximab group) − 44.4 ± 9.4 y.o. (placebo group)	- TRD (MGH-S ≥2, QIDS-SR-16 > 14)	Comparison symptoms improvement between placebo vs infliximab group (HDRS-17, CGI-S) and association of inflammatory biomarkers with treatment response (hs-CRP, TNF-α and its soluble receptor)	No difference between groups for HDRS-17 scores – a tendency for interaction between treatment and hs-CRP	- Autoimmune disorders - History of tuberculosis - A currently active fungal, bacterial or viral infection - History of cancer excluding eradicated basal or squamous cell carcinoma of the skin - Unstable cardiac, endocrine, hepatic, renal or neurologic disease - History of schizophrenia or other active psychotic disorders - Substance abuse disorder - Current suicide ideation	USA
Savitz et al., 2015a	Cross-sectional study	128	− 49 current MDD - Mean age 35.4 years (SD ± 9.8), 78% female; − 21 remitted MDD - Mean age 30.8 years (SD ± 12.2), 57% female − 58 HC - Mean age 32.8 years (SD ± 10.7), 57% female	MDD	Evaluate eventual differences in TRP, KYN, KYNA, 3HK, QA, CRP, IL-1RA between study groups	- Lower levels of KYNA/QA in MDD vs HC - Inverse correlation for KYNA/QA and anhedonia in current MDD episode - Negative correlation for lifetime number of MDD episodes and KYNA/QA, positive correlation for months in remission and KYNA/QA	- Serious suicidal ideation or behavior - Concomitant medical therapy - Cardiovascular, respiratory, endocrine, and neurological diseases - Drug abuse in the six months or dependence one year prior enrollment	USA
Savitz et al., 2015b	Cross-sectional study	49	− 29 MDD Mean age 36.4 ± 10.0 years, 83% female − 20 HC Mean age 35.0 ± 10.9 years, 52% female	MDD Biomarkers: serum BDNF, IL-6, CRP, and IL-1RA	- KA/QA ratio trended lower in MDD vs HC - KA/QA negative correlation with anhedonia but positive with total hippocampal and amygdala volume in MDD - Higher IL-1RA, QA and KYN in MDD - KA, TRP and KYN positive correlated with hippocampal and amygdala volumes in MDD	- Higher hippocampal and amygdala volumes in MDD patients associated with higher KA/QA	- Serious suicidal ideation or attempts - General MRI exclusion criteria - Drug or alcohol abuse history six months before enrollment or one year before enrollment for substance dependence	USA
Tuglu et al., 2003b	Experimental study	43	− 26 MDD (39.38 ± 14.5 y.o.) − 17 HC (37.1 ± 11.0 y.o.) - Female 60%	BDI HDRS Biomarkers: serum TNF-a - serum CRP - Leukocyte count	Evaluate the association of selected biomarkers with treatment response in MDD	- Higher TNF-a and leukocyte count at baseline for MDD vs HC - Normalization of inflammatory markers after 6-weeks treatment with SSRI	- Any Axis I or II diagnoses - Pregnancy - Acute or chronic diseases in the three months prior enrollment	TUR

*Abbreviations: 3HA - 3-hydroxyanthranilic acid; 3HKYN - 3-hydroxykynurenine; AA - anthranilic acid; AGO – Agoraphobia; ALSPAC - Avon Longitudinal Study of Parents And Children; ASD – Autism Spectrum Disorders; AUS – Australia; BAI – Beck Anxiety Inventory; BDI – Beck Depression Inventory; BHS – Beck Hopelessness Scale; BLE – Brief Life Event Scale; BLG – Belgium; BSI – Beck Suicide Ideation; CAPS – Clinician-Administered PTSD scale; CGI-S - Clinical Global Impression Scale – Severity; CSF – Cerebrospinal Fluid; CCL11 - Eotaxin-1; DEN – Denmark; EST – Estonia; FG – Fasting Glucose; FGF - fibroblast growth factor; HC – Healthy Controls; HDL – HDL cholesterol; HDRS – Hamilton Depression Rating Scale; GAS – Goldberg Anxiety Scale; G-CSF - Granulocyte Colony Stimulating Factor; GAD – General Anxiety Disorder; GDS – Geriatric Depression Scale; GER – Germany; GM-CSF - Granulocyte Macrophage Colony Stimulating Factor; HADS- Hospital Anxiety and Depression Scale; hs-CRP – High Sensitivity C-Reactive Protein; IDS -SR – Inventory of Depressive Symptomatology Self-Report; IL-n – Interleukin; IL-1 – Interleukin 1;* IL-6 – Interleukin 6; *IL-*1RA *– Interleukin receptor Antagonist; Interferon gamma-induced protein 10 - Interferon gamma-induced protein 10; IRN – Iran; ITA – Italy; KYNA - kynurenic acid; MADRS - Montgomery-Åsberg Depression Rating Scale; MAOIs – Monoamine oxidase inhibitors; MCP1/CCL2 - Monocyte chemoattractant protein-1; MCP-1 -* monocyte chemotactic protein; *MMSE – Mini Mental State Examination; MGH-S – Massachusetts General Hospital – Score; MGHATRQ – Massachusetts General Hospital Antidepressant Treatment Response Questionnaire; MIP-1α/CCL3 - Macrophage inflammatory protein-1 alpha; MIP-1β/CCL4 - Macrophage inflammatory protein-1 beta; NESDA - Netherlands Study of Depression and Anxiety; PD – Panic Disorder; PDGF-B - Platelet-Derived Growth Factor Beta; Post DEX – Post Dexamethasone; PSS – Perceived Stress Scale; PTSD – Post Traumatic Stress Disorder;* QA – Quinolinic Acid; *RANTES/CCL5 - Regulated on Activation Normal T Cell Expressed and Secreted; RCT – Randomized Controlled Trial; RLCQ – Recent Life events questionnaire; SHAPS – Snaith Hamilton Pleasure Scale; SP – Social Phobia;* SSRI – Selective Serotonin Reuptake Inhibitors; STAI – Spielberg State Trait Anxiety Rating Scale; sTNF-αR2 – Soluble Tumor Necrosis Factor receptor 2; *sVCAM-1 - serum vascular cell adhesion molecule-1; TNF-α – Tumor Necrosis Factor α; TRD – Treatment Resistant Depression; TRP – Tryptophan; TUR – Turkey; TRYG – Triglycerides; UK – United Kingdom; VEGF - Vascular Endothelial Growth Factor; y.o. – years*.

### 4.5 Growth factor and neurotrophins

Daskalakis and colleagues have analyzed the transcriptomic profile within the blood and key-stress sensitive brain areas (i.e. amygdala and hippocampus) in PTSD-susceptible and resilient rats, and they found that 22 genes encoding for growth factors were differentially expressed, and 5 of them (angiotensinogen, epidermal growth factor, fibroblast growth factor 2, nerve growth factor, TGF-β1) were convergent across tissue Daskalakis et al., 2016. Among the neurotrophic factors, preclinical data indicate a prominent role for brain-derived neurotrophic factor (BDNF) as a biomarker for stress vulnerability and psychiatric disorders (Nestler et al., 2002; Miller et al., 2017). Stress exposure reduces BDNF expression in rodent models leading to high susceptibility for the development of stress-related disorders (Stepanichev et al., 2014). Prenatal stress has been shown to reduce BDNF expression in several stress-related brain areas at weaning and adulthood in rats, altering then neuronal plasticity and possibly leading to increased vulnerability for psychiatric disorder development (Boersma et al., 2014; Luoni et al., 2014). A common single-nucleotide polymorphism in the *BDNF* gene, a methionine (Met) substitution for valine (Val) at codon 66 (Val66Met), is associated with alterations in brain anatomy and memory. Typically, Met carriers show less activity-dependent release of the BDNF protein in the hippocampus than Val homozygotes (Egan et al., 2003; Hashimoto et al., 2008) and studies in rodents have established that augmenting BDNF in limbic brain regions pathway facilitates extinction of traumatic memory (Peters et al., 2010; Andero et al., 2012). Additionally, evidence from BDNF^Met/Met^ mice indicate that, when placed in stressful settings, Met carriers exhibit increased anxiety-related behaviors (Chen et al., 2006). This would provide a neurobiological basis as to why Met allele carriers would be more vulnerable to develop stress- and trauma-related disorders (Zhang et al., 2016). Converging evidence from animal and human studies also indicate this BDNF gene polymorphism as a predictor for clinical presentation in schizophrenia (Farcas et al., 2023; Buhusi et al., 2023). Table 4 summarizes the results for the included paper reporting on clinical model for neurotrophins biomarkers.

**Table 4:** Study selection of clinical model of neurotrophin biomarkers.

Author, year	Study design	Sample size	Sample characteristics	Diagnostic category or clinical correlate	Main outcomes reported	Main results	Exclusion criteria	Country
Hashimoto et al., 2008	Cross-sectional study	58	- mean age 36.4 ± 10.6 y.o. - Homozygous Val-BDNF (n = 17, female 70.5%) - Val/met BDNF group (n = 29, female 82.7%) - Homozygous Met-BDNF (n = 12, female 83.3%)	Val66Met polymorphism of BDNF on hippocampal-related memory activity - WAIS-R - WMS-R	Assess the effect of selected BDNF genotype on clinical assessment and fMRI measurements on hippocampal activity	- Negative correlation between the dose of the Met-BDNF and encoding-related brain activity in bilateral hippocampi and right parahippocampal gyrus - No effect of genotype of episodic memory on behavioral assessment or retrieval-related brain activity	- Medical conditions affecting the central nervous system - Psychiatric conditions - Substance abuse or dependence - Chronic lung disease - Kidney disease - Chronic hepatic disease - Diabetes mellitus - Substance abuse or dependence - Atypical headache - head trauma with loss of consciousness	JAP

Abbreviations:fMRI – functional Magnetic Resonance Imaging; JAP – Japan; WAIS-R Wehsler Adult Intelligence Scale-Revised; WMS-R – Wechsler Memory Scale-Revis.

### 4.6 Sleep biomarkers

In rodent studies, both direct (altered sleep properties) and indirect (neural pathways involved in sleep) alterations have been identified as potential biomarkers of vulnerability/resilience to stress-related disorders. Recent evidence has reported that two altered non-rapid eye movement (NREM) stages (N–S3 and N–S1) allow to determine which rat will develop vulnerability for depressive-like phenotype before and after stress exposure (Claverie et al., 2023). By using polysomnographic recordings, Monari and colleagues found that genetically modified rat lines with low corticosterone responsiveness (and more vulnerability to developing PTSD-like symptoms) exhibited less time in REM stages during the inactive state and more time during the awake state and that noradrenergic release is involved in these effects (Monari et al., 2023). Alterations in sleep (e.g., REM sleep, transition to REM sleep, waking as well as theta and sigma band power) have also been found to be linked to fear memory alterations in an animal model of PTSD (Vanderheyden et al., 2015). Table 5 summarizes the main finding of the study selected among papers reporting on sleep biomarkers.

**Table 5:** Study selection for clinical models of sleep biomarkers.

Author, year	Study design	Sample size	Sample characteristics	Diagnostic category or clinical correlate	Main outcomes reported	Main results	Exclusion criteria	Country
Floam et al., 2014	Cross-sectional study	49	- Female 65.3% - INS-mean age 25.3 ± 1.6 y.o. - CON- mean age 25.4 ± 1.4 y.o.	Insomnia disorder - PSQI - LOT-R - STAI Biomarkers: urinary overnight NEP - Monocyte count, plasma IL-6 serum CRP - Serum cortisolr	Evaluate the association of selected biomarkers with insomnia disorder and other psychometric assessments	INS group had higher inflammation and HPA composite scores	- Axis I disorders - Medical disorders, including those associated with sleep disorders other than insomnia - Medications other than contraceptives	USA

Abbreviations:CRP – C reactive protein; IL-6 Interleukin −6; NEP – Norepinephrine; PSQI – Pittsburgh Sleep Quality Index; LOT-R – Life Orientation Test-Revised; STAI – State Trait Anxiety Inventory.

### 4.7 Digital biomarkers

The use of digital biomarkers in preclinical studies started in the last few years when software-based video technologies allowed to measure numerous behavioral and physiological parameters (e.g., time spent in specific zones, total distance travelled, velocity; electromyography (EMG) for heart rate measurements) have been introduced (Marsicano et al., 2002; Reich et al., 2013; Martin et al., 2002; Xing et al., 2011). Nowadays, these systems have evolved even further, making it possible to do an automated behavioral analysis as they are able to recognize more specific behaviors (e.g., grooming, rearing, and wall-rearing, 145,146). The refinement of these techniques will allow the development of useful non-invasive tools to be integrated with conventional established biomarkers and allow faster identification of subjects susceptible to stress-related diseases. Further underscoring the potential that these technologies may hold for the future, a 2019 study reported on 26 volunteers wearing commercially-available wearables, finding that a low-within subject resting heart rate appears to be associated with the effects of stress and mental exhaustion (de Vries et al., 2021). If such early evidence were to be confirmed in larger and longer studies, digital environmental and both intrinsic and extrinsic individual biomarkers may further enrich our analyses. Table 6 summarizes the main finding of the study selected among paper reporting on digital biomarkers.

**Table 6:** Study selection for clinical models of digital biomarkers.

Author, year	Study design	Sample size	Sample characteristics	Diagnostic category or clinical correlate	Main outcomes reported	Main results	Exclusion criteria	Country
de Vries et al., 2021	Prospective follow-up study	26 interns	− 19.2-33.2 y.o., female 92.3%	EMA (HRV, TST, perceived demands, stress, mental exhaustion) for 15 weeks	Evaluate whether HRV and TST could be indicative and predictive of withi-day accumulation of negative consequences of stress and mental exhaustion	- Resting HRV appeared to buffer against the positive association between demands and stress and between stress and mental exhaustion - Stress did not impact on TST - Mental exhaustion negatively predicted HRV the following morning	N/A	NET

Abbreviations: EMA – Ecological Momentary Assessment; HRV – Hear Rate Variability at rest Polar H7 Bluetooth Chest Strap; NET – Netherlands; TST – Total Sleep Time.

## 5 Discussion and future directions

The ever-growing body of literature surrounding the dyadic interaction of stress and resilience further supports the notion that integrating biomarkers paradigms in developing risk-resilience theories is paramount for the future development of preclinical and clinically viable models. In this setting, our review suggests that considerable attention has been devoted to the HPA and its association with derangements of the immune system activation in both preclinical and clinical models. Cytokines and stress hormones may be at the crossroads of major central- and peripheral regulating pathways and, therefore, represent a particularly promising area for the development of functional biomarkers and novel therapeutic avenues. For example, HPA derangements and different sensitivity levels to glucocorticoids have been reported in PTSD samples, albeit not uniformly, suggesting the possible significance of stress response in the development of the disorder (Yehuda et al., 2015). Lower levels of glucocorticoid expression may, in turn, lead to higher expression of pro-inflammatory proteins, such as INF-γ, IL-1β, TNF-α through their impact on gene expression (Passos et al.). Numerous papers included in this review reported on the association between MDD and inflammatory markers. Though a clear involvement of immune alterations in the onset and treatment of MDD has emerged, our comprehension of such an intricate link remains limited. Recently, it has been proposed that this interplay may be mediated by neural and behavioral plasticity (Branchi et al., 2024). Plasticity -- that is, the capability of the brain and behavior to be modified according to contextual factors -- is recognized as fundamental in psychiatry and mental health since it plays a crucial role in the reorganization of neural circuits and behavioral outcomes during the transition from psychopathology to wellbeing (Branchi, 2022; Duman et al., 1999; Lindenberger et al., 2017; Price and Duman, 2020). However, plasticity is not good per se (Branchi, 2011; Delli Colli et al., 2022). While plasticity renders the brain more susceptible to change, the ultimate outcome of such change is not determined by plasticity but by contextual factors, including living conditions and psychotherapeutic interventions (Branchi, 2011; Belsky et al., 2009). Accordingly, it has been shown that treatments enhancing plasticity have a more beneficial outcome when combined with supportive environmental conditions (Bottemanne et al., 2022; Carhart-Harris et al., 2018; Chiarotti et al., 2017; Lepow et al.; Viglione et al., 2019). Recent studies have shown that deviations towards either extreme immune activation or suppression disrupt the molecular mechanisms involved in neural plasticity (Golia et al., 2019; Hewett et al., 2012; Santello and Volterra, 2012; Yirmiya and Goshen, 2011). Consequently, a pro-inflammatory profile triggered by various factors, including stress or immune and metabolic disorders, has been associated with impaired plasticity. This, in turn, hinders the potential for recovery (Branchi et al., 2024). Therefore, depressed patients experiencing chronic high inflammatory levels have been hypothesized to remain confined within their psychopathology. This condition has been referred to as the *inflammatory trap of depression* (Branchi et al., 2024). In these patients, the normalization of immune activation is a critical step to reinstate plasticity. However, since plasticity does not lead to improvement per se but increases the likelihood of recovery, immunomodulatory treatments should be combined with contextual factors, such as environmental interventions or psychotherapies, to achieve well-being and promote resilience. Accruing evidence points to the potential of harnessing technological advancement in interpreting the gene-environment interaction by assessing gene expression level, as this may represent a viable strategy for encompassing both heredity and environment (Tylee et al., 2015). Past reports have suggested the possible worth of probing DNA methylation by assessing its association with suicide ideation intensity (Al-Chalabi et al., 2023). Post-mortem analysis comparing suicide victims with controls appears to support such findings (Kouter et al., 2019; McGowan and Szyf). Overall, the available evidence suggests a role for the involvement of astrocytes, stress response, microglia and the immune system that collectively may influence the overall risk for suicide, further underscoring the possible utility of such an approach in enlarging our understanding of complex clinical outcomes (Piras et al., 2022). On a similar line of reasoning, prenatal maternal health has been recognized as a significant determinant of public health with its long-term impact on development and health, including the risk of developing psychopathology (Meaney, 2018). In a 2022 paper, Kee et al. 2022 described sex-specific differences in the genome-wide DNA methylation, with prenatal maternal depressive symptom levels associated with maternal methylome only among mothers of female fetuses and with evidence of female-specific interactions with fetal-facing placenta methylome. This maternal-fetal interaction may represent a mechanism of intergenerational transmission for the methylation profile. Interestingly, these findings were replicated in two different cohorts totaling 878 mothers of Chinese (n = 491) and Caucasian ethnicity (n = 387, 278). Arguably, for further development of the field, it will be critical to have a growing level of attention devoted to defining clinically relevant outcomes and how to realistically test their potential association with tested biomarkers in appropriately tailored study design. In this regard, the future applications of environmental, digital biomarkers have the potential to inform future diagnostic paradigms and integrate clinical assessment (Adler et al., 2022).

## 6 Conclusions

Notwithstanding significant progress in the field, the clinical applications of stress-resilience biomarkers appear out of reach for the foreseeable future due to methodological issues in clinical phenotyping, preclinical model correlation to human pathology and the inherent difficulties in studying end outcomes over a lifetime following stress exposure. A widening rift has opened between preclinical and clinical applications across all fields of medicine. Increased integration of preclinical, clinical translational models more strictly rooted in empirically-based phenotype and, significantly, clinical findings feedbacking in preclinical model development could be a promising avenue in trying to bridge this gap.

## Role of funding sources

This paper represents a spontaneous initiative of the ECNP's Resilience network, which received no specific funding. We acknowledge the support of funds by the European Union - Next Generation EU - NRRP M6C2 - Investment 2.1 Enhancement and strengthening of biomedical research in the NHS, project titled “Reli€ving the burden of Post-Traumatic Stress Disorder: disentangle mechanisms of vulnerability and resilience to tailor personalized therapeutic intervention (PNRR-MAD-2022-12376156)”. The funder had not role in the development of this paper.

## Declaration of competing interest

The authors declare that they have no known competing financial interests or personal relationships that could have appeared to influence the work reported in this paper.
